# Numerical Study of 3D MHD Mixed Convection and Entropy Generation in Trapezoidal Porous Enclosure Filled with a Hybrid Nanofluid: Effect of Zigzag Wall and Spinning Inner Cylinder

**DOI:** 10.3390/nano12121974

**Published:** 2022-06-08

**Authors:** Apichit Maneengam, Tarek Bouzennada, Aissa Abderrahmane, Kaouther Ghachem, Lioua Kolsi, Obai Younis, Kamel Guedri, Wajaree Weera

**Affiliations:** 1 Department of Mechanical Engineering Technology, College of Industrial Technology, King Mongkut’s University of Technology North Bangkok, Bangkok 10800, Thailand; apichit.m@cit.kmutnb.ac.th; 2 Mechanics of Materials & Plant Maintenance Research Laboratory (LR3MI), Mechanical Engineering Department, Faculty of Engineering, Badji Mokhtar University, Annaba 23052, Algeria; tarek.bouzennada@univ-annaba.org; 3 Laboratoire de Physique Quantique de la Matière et Modélisation Mathématique (LPQ3M), University Mustapha Stambouli of Mascara, Mascara 29000, Algeria; a.aissa@univ-mascara.dz; 4 Department of Industrial Engineering and Systems, College of Engineering, Princess Nourah bint Abdulrahman University, Riyadh 11671, Saudi Arabia; kgmaatki@pnu.edu.sa; 5 College of Engineering, Mechanical Engineering Department, Ha’il University, Ha’il City 81481, Saudi Arabia; l.kolsi@uoh.edu.sa; 6 Laboratory of Meteorology and Energy Systems, University of Monastir, Monastir 5000, Tunisia; 7 Department of Mechanical Engineering, College of Engineering at Wadi Addwaser, Prince Sattam Bin Abdulaziz University, Al-Kharj 11991, Saudi Arabia; oubeytaha@hotmail.com; 8 Mechanical Engineering Department, College of Engineering and Islamic Architecture, Umm Al-Qura University, Makkah 21955, Saudi Arabia; kmguedri@uqu.edu.sa; 9 Department of Mathematics, Faculty of Science, Khon Kaen University, Khon Kaen 40002, Thailand

**Keywords:** magneto-hydrodynamics, hybrid nanofluid, mixed convection, entropy generation, porous media

## Abstract

A numerical study was performed to analyze the impact of the combination of several factors on heat transfer rate, flow behavior, and entropy generation in a hybrid nanofluid occupying a porous trapezoid enclosure containing a rotating inner tube. The governing equations were discretized and solved using the Finite Element Method using Comsol multiphysics. The effects of the Darcy and Hartman number, nanoparticle volume fraction (from 0 to 6%), the utilization of various zigzag patterns of the hot wall, and the rotation speed of the inner tube (Ω = 100. 250 and 500) are illustrated and discussed in this work. The outputs reveal that flow intensity has an inverse relationship with Hartman number and a direct relationship with the Darcy number and the velocity of the inner tube, especially at high numbers of undulations of the zigzag hot wall (N = 4); also, intensification of heat transfer occurs with increasing nanoparticle volume fraction, Darcy number and velocity of the inner tube. In addition, entropy generation is strongly affected by the mentioned factors, where increasing the nanoparticle concentration augments the thermal entropy generation and reduces the friction entropy generation; furthermore, the same influence can be obtained by increasing the Hartman number or decreasing the Darcy number. However, the lowest entropy generation was found for the case of Ø = 0, Ha = 0 and Da = 0.01.

## 1. Introduction

Convective heat transfer and entropy generation have attracted a great deal of attention in recent years, because they are relevant for several engineering applications, such as chemical engineering processes and lubrication, as well as in the design of solar collectors, heat exchangers, nuclear reactors, and cooling electronic chips [1,2], in addition to many other domains. For instance, recently, carbon nanoparticles such as graphene, graphite, and other metals like magnesium and molybdenum have become the best choice for storing hydrogen, with these compounds having immense energy storage capacity and high hydrogenation and dehydrogenation rates [3]. Many techniques have been utilized to assist heat distribution in the fluid, such as the addition of highly thermally conductive nanoparticles (ordinary/hybrid compounds), metal foam, rotating cylinders inside the enclosures [4,5,6,7], among others. Thus, scientists are seriously seeking to enhance heat transfer performance; however, regardless of the importance of heat transmission by conduction or radiation, heat transfer by convection has attracted the greatest attention. 

Many previous works have taken nanofluid mixed convection as their subject. Selimefendigil and Öztop [7] studied mixed convection numerically in a lid-driven trapezoidal enclosure filled with Al_2_O_3_–water nanofluid under the effect of an external magnetic field. The upper and bottom walls are assumed to be at cold and hot temperatures, respectively; while the two side walls are insulated, the upper wall slides horizontally. The authors mentioned that the mean Nusselt number increases with increasing Richardson number, magnetic force, and solid element volume fraction. Chamkha et al. [8] carried out a numerical study to elucidate the effect of the Darcy number on heat transfer in a square cavity with a rotating inner cylinder. The results revealed that the heat transfer was better when the angular velocity of the cylinder was higher. Additionally, the maximum enhancement in heat transfer was obtained when Darcy number was lower. Roslan et al. [9] conducted a numerical study in a square cavity filled with a Nanofluid. The study analyzed the effect of several factors on heat transfer, while varying the type of nano-additive, the rotating speed of an inner cylinder, and its radius. The outcomes showed that the best enhancement in heat transfer was obtained when using the smallest cylinder, a faster angular cylinder speed, and high-conductivity nano-additives. Al-Farhany and Abdulsahib [10] numerically investigated how much the heat performance of a nanofluid would be affected when changing the concentration of nanocompounds, Darcy number, and the rotation speed of a cylinder involved inside a square enclosure. The authors concluded that the Nusselt number reached a peak value when increasing the cylinder rotation speed and the Darcy number. Additionally, Ghasemi and Siavashi [11] investigated the effect of adding Cu-nanoparticles to water in a porous enclosure on heat transfer during the presence of magnetic force. They found that low Darcy numbers result in stagnation of the convection effect, but by increasing Darcy numbers, both conduction and convection are improved, while adding nanoparticles also positively affects heat transfer; on the other hand, the magnetic force always has a negative effect on Nu

Additionally, many research works have highlighted the impact of the shape of the enclosure on the thermal and flow behaviors. Hussein et al. [12] carried out a numerical study investigating the impact of a trapezoidal capsule with a sinusoidal bottom wall on both the fluid flow and heat transfer advancement of a nanofluid. The selected nanofluid was CuO–water, which occupies two separate regions, the bottom region is saturated with porous media, and the top region remains free. The results indicated enhancements in terms of increased Darcy/Rayleigh numbers, nanoparticle concentration, inner tube radius, and rotation speed; however, an inverse effect was observed with increased Darcy number and undulation of the hot bottom wall. Pal et al. [13] numerically investigated the thermal transmission in mixed convection mode for Cu–water nanofluid occupying a wavy heated bottom wall cavity, where both the walls on both the left and right sides were maintained at a cold temperature while the top adiabatic wall was sliding horizontally. The results indicated that the wavy hot wall had a significant effect when both the nanocompounds and fluid conductivities were equivalent. Cho [14] carried out a numerical study to predict the heat distribution rate within a copper–water nanofluid in mixed convection mode inside a wavy-wall cavity. The fluid received heat from a sliding left-hand-side wall, while the right wall was cold and wavy, and the remaining walls were isolated. It was observed that that the average Nusselt number reached a high value with increasing nanoparticle volume fraction, wavy surface amplitude, and Richardson and Reynolds numbers. Mekroussi et al. [15] numerically studied the effect of the inclination angle on heat transfer performance during the mixed convection of fluid inside a sinusoidal wavy lid-driven enclosure. The outcomes indicated that the average Nusselt number increased with increasing numbers of undulations of the bottom wall or with increasing cavity inclination angle.

Recently a new category of nanofluids, called hybrid nanofluids, has become a trend in heat transfer enhancement, and several studies have discussed the effectiveness of this type of nanofluid. Cimpean et al. [2] presented a numerical study discussing the role of mixing nanoparticles of Cu and Al_2_O_3_ with water (selected as hybrid nanofluid); the study was performed in a porous trapezoidal cavity, assuming that the top wall slid horizontally and was cold, while the bottom wall and the two side walls were respectively hot and adiabatic. The results illustrated that for Da ≤ 10^−3^, the conduction mode dominated whenever Cu nanoparticle concentration was higher, with the same reduction in the concentration of Al_2_O_3_ nanoparticles; however, the opposite effect was obtained for Da > 10^−3^. Jarray et al. [16] described how heat transfer was influenced inside a horizontal rectangular porous chamber filled with Ag-MgO–water hybrid nanofluid. The numerical outcomes revealed that the improvement in heat transfer was specifically dependent on the Darcy number for certain nanoparticle concentrations, where the heat transfer rate increased with increasing nanoparticle volume fraction for weak permeability (Darcy) until reaching a peak rate, and then dropped sharply at high nanoparticle concentrations; conversely, at high values of permeability, the heat transfer rate was enhanced with any amount of nanoparticles. 

Additionally, the effect of the shape of the nanoparticles has been investigated. Ghadikolaei and Gholinia [3] numerically investigated the effect of nanoparticle shape on heat transfer in a 3D configuration containing a hybrid nanofluid. The output data proved that the heat transfer rate increased with increasing nano additive shape factor.

Moreover, many previous works have considered entropy generation, which arises from several factors. Aich [17] carried out a three-dimensional numerical study of free convection with entropy generation of air inserted into a triangular locale. The fluid received heat from an isothermal heater placed in the bottom section, while all of the walls were adiabatic except the inclined wall, which was assumed to be cold. It was found that the heat transmission improved as the Rayleigh number increased; in addition, there was a direct relation between Rayleigh number values and entropy generation. Kolsi [18] investigated the effects of Richardson and Hartmann numbers on heat transfer and entropy generation in a fluid placed in a 3D cubical cavity. The outcomes showed that the Bejan and mean Nusselt numbers stood in an inverse relationship with Ha and Ri numbers; additionally, increasing Ha caused the entropy generation rate to drop. Hamzah et al. [19] studied the influence of Richardson and Darcy numbers on heat transfer and entropy generation in a wavy lid-driven porous container filled with CNT–water nanofluid. The results indicated that the mean Nusselt number was positively affected by the increased amplitude of the wavy wall, as well as Richardson and Darcy numbers. Simultaneously, the entropy generation rate decreased. Nguyen et al. [20] carried out a numerical study in three different channels; the work aimed to determine the impact of the magnetic field on the heat transfer and entropy generation of FMWNT–water (functionalized multi-walled carbon nanotubes). The results indicated that the magnetic force with this type of nanofluid played a positive role in enhancing heat transfer and decreasing entropy generation. Barnoon et al. [21] numerically examined the role of Hartman and Richardson numbers in enhancing heat distribution and reducing the entropy generation in a lid-driven container with rotating cylinders filled with a nanofluid. It was found that reducing both Ha and Ri led to improved heat transfer and reduced entropy generation. Comprehensive studies in which more outcomes of nanofluids/hybrid nanofluids are described can be found in [22,23,24,25,26]. 

On the basis of the literature review presented above, as well as other available research works, the present work is a theoretical proposal that would be beneficial in several engineering domains. This study aims to explore the effects of combining the use of hybrid nanofluids (water–MoS_2_) at different G.O. concentrations (0, 3, and 6% of MoS_2_/G.O.) and a never-before-used zigzag-patterned wall in a 3D trapezoidal porous enclosure containing a rotating tube under magnetic force. The results are presented in terms of isothermal surfaces, streamlines (arrow-spaced streamlines), total entropy generation contours, and mean Nusselt and Bajan numbers.

## 2. Studied Configuration and Mathematical Formulation 

**Figure 1:**
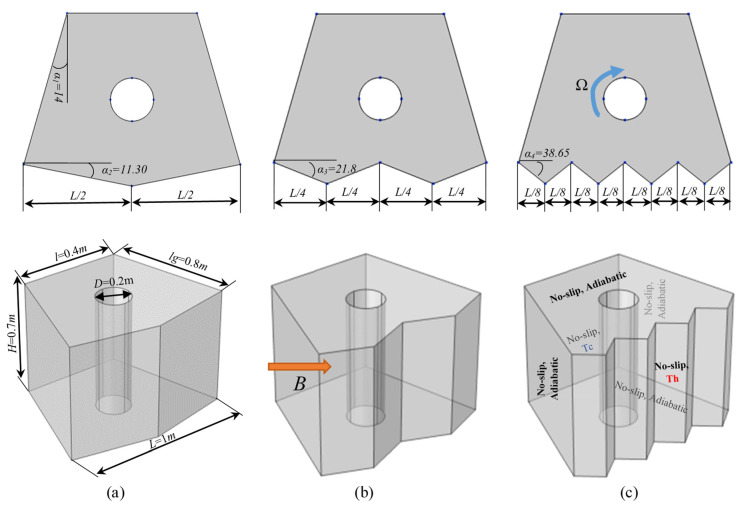
The computational domain and the boundary conditions: 3D view of the enclosure (bottom) and 2D view of the geometry with the undulation pattern illustrations of the zigzag wall (top). (**a**) N = 1, (**b**) N = 2, and (**c**) N = 4.

The thermophysical properties of water, MoS_2_, and G.O. are listed in Table 1. The configuration considered in this work is illustrated in Figure 1. It consists of a porous 3D trapezoidal cavity containing a hybrid nanofluid (water with MoS2 and G.O. nanoparticles), with a magnetic force applied along the positive y- and z-directions. All walls are assumed to be adiabatic and non-slipping except the zigzag wall, which is considered to be at the hot temperature Th, while the front wall is considered to be at the cold temperature Tc. The zigzag wall is taken to be the main geometric influencer, and has different numbers of undulations (various peak numbers, N = 4, 2, and 1). A rotating central tube is positioned at the center of the cavity to play an active role in the fluid movement alongside the passive effect of buoyancy force (mixed convection).

### 2.1. Mathematical Model

By assuming that the study takes place within a porous 3D cavity and that the selected liquid is a Newtonian incompressible fluid subject to a laminar regime, the governing equations are as follows [27,28].

The conservation equations:

(1) $$\frac{\partial U}{\partial X}+\frac{\partial V}{\partial Y\;}+\frac{\partial W}{\partial Z}=0$$

The momentum equations:

(2) $$\begin{array}{l} \frac{\rho_{hnf}}{\rho_{f}}\left[\frac{U}{\varepsilon^{2}}\frac{\partial U}{\partial X}+\frac{V}{\varepsilon^{2}}\frac{\partial U}{\partial Y}+\frac{W}{\varepsilon^{2}}\frac{\partial U}{\partial Z}\right]=\\ \;-\frac{\rho_{hnf}}{\rho_{f}}\frac{\partial P}{\partial X}+\frac{1}{Re}\frac{1}{\varepsilon }\frac{\mu_{hnf}}{\mu_{f}}\left(\frac{\partial U}{\partial X}+\frac{\partial U}{\partial Y}+\frac{\partial U}{\partial Z}\right)-\frac{\mu_{hnf}\;\;\;}{\mu_{f}Re\mathrm{Da}}U-\frac{\rho_{hnf}}{\rho_{f}}\frac{0.55}{\sqrt{\mathrm{Da}}}\sqrt{U^{2}+V^{2}+W^{2}}\;U \end{array}$$

(3) $$\begin{array}{l} \frac{\rho_{hnf}}{\rho_{f}}\left[\frac{U}{\varepsilon^{2}}\frac{\partial V}{\partial X}+\frac{V}{\varepsilon^{2}}\frac{\partial V}{\partial Y}+\frac{W}{\varepsilon^{2}}\frac{\partial V}{\partial Z}\right]=\\ \;-\frac{\rho_{hnf}}{\rho_{f}}\frac{\partial P}{\partial Y}+\frac{1}{Re}\frac{1}{\varepsilon }\frac{\mu_{hnf}}{\mu_{f}}\left(\frac{\partial V}{\partial X}+\frac{\partial V}{\partial Y}+\frac{\partial V}{\partial Z}\right)-\frac{\mu_{hnf}\;\;\;}{\mu_{f}Re\mathrm{Da}}V-\frac{\rho_{hnf}}{\rho_{f}}\frac{0.55}{\sqrt{\mathrm{Da}}}\sqrt{U^{2}+V^{2}+W^{2}}V-\frac{\sigma_{hnf}}{\sigma_{f}}Ha^{2}\;\frac{V}{\varepsilon } \end{array}$$

(4) $$\begin{array}{l} \frac{\rho_{hnf}}{\rho_{f}}\left[\frac{U}{\varepsilon^{2}}\frac{\partial W}{\partial X}+\frac{V}{\varepsilon^{2}}\frac{\partial W}{\partial Y}+\frac{W}{\varepsilon^{2}}\frac{\partial W}{\partial Z}\right]=-\frac{\rho_{hnf}}{\rho_{f}}\frac{\partial P}{\partial Z}+\frac{1}{Re}\frac{1}{\varepsilon }\frac{\mu_{hnf}}{\mu_{f}}\left(\frac{\partial W}{\partial X}+\frac{\partial W}{\partial Y}+\frac{\partial W}{\partial Z}\right)\\ \;-\frac{\mu_{hnf}}{\mu_{f}Re\mathrm{Da}}W-\frac{\rho_{hnf}}{\rho_{f}}\frac{0.55}{\sqrt{\mathrm{Da}}}\sqrt{U^{2}+V^{2}+W^{2}}\;W+\frac{{\left(\rho \beta \right)}_{hnf}}{{\left(\rho \beta \right)}_{f}}Ri\theta -\frac{\sigma_{hnf}}{\sigma_{f}}Ha^{2}\;\frac{W}{\varepsilon } \end{array}$$

where *U*, *V*, *W* and *P* are the dimensionless velocity components in the *X*, *Y*-, and *Z*-directions and the dimensionless pressure, respectively; *ρ* *µ*, *σ*, *β* and *k* are the density, dynamic viscosity, electrical conductivity, thermal expansion coefficient, and thermal conductivity of the material, respectively; *ε* is the matrix porosity; and the *hnf* and *f* symbols refer to the hybrid nanofluid and the base fluid (water), respectively.

The last term in Equations (3) and (4) are Lorentz force [27].

The heat equation:

(5) $$U\frac{\partial \theta }{\partial X}+V\frac{\partial \theta }{\partial Y}+W\frac{\partial \theta }{\partial Z}=\frac{{\left(\rho c_{P}\right)}_{f}}{{\left(\rho c_{P}\right)}_{hnf}}\frac{k_{eff}}{k_{f}}\frac{1}{Re\mathrm{Pr}}\left[\frac{\partial^{2}\theta }{\partial X^{2}}+\frac{\partial^{2}\theta }{\partial Y^{2}}+\frac{\partial^{2}\theta }{\partial Z^{2}}\right]$$

where *θ* is the dimensionless temperature and *k_eff_* is the effective conductivity, which is determined by $k_{eff}=(1-\varepsilon )k_{s}+\varepsilon k_{hnf}$ (*k_s_* is the thermal conductivity of the solid matrix forming the porous layer, *k_s_* = 0.78 W·m^−1^·K^−1^ and ε = 0.37)

$$X,Y,Z=\frac{x,y,z}{L},\;U,V,W=\frac{\left(u,v,w\right)L}{\alpha_{nf}},\;\theta =\frac{T-T_{c}}{T_{h}-T_{c}},\;P=\frac{pL^{2}}{\rho_{f}\alpha_{f}^{2}},\mathrm{Pr}=\frac{v_{f}}{\alpha_{f}},\;\mathrm{Da}=\frac{\kappa }{L^{2}}$$

$$Ra=\frac{g\beta_{f}(T_{h}-T_{c})L^{3}}{\alpha_{f}v_{f}},\;\mathrm{Ha}=LB\sqrt{\frac{\sigma_{hnf}}{\mu_{hnf}}},\;\varepsilon \mathrm{is}\;\mathrm{the}\;\mathrm{porosity},\;Ri=\frac{Ra}{\mathrm{Pr}Re^{2}}=\frac{Ra\mathrm{Pr}}{\Omega^{2}R^{2}},\;Re=\frac{\Omega R}{\mathrm{Pr}},\;R=\frac{r}{L},\;\mathrm{and}\;\Omega =\frac{\omega L^{2}}{\alpha_{nf}}$$

where *α_f_*, and *ν_f_* are the thermal diffusivity and kinematic viscosity of water, respectively, *κ* is permeability, *g* is gravity acceleration, *B* is the magnetic field and *r* is the radius of the cylinder, *ω* and Ω are the angular rotational velocity and dimensionless angular rotational velocity, respectively, and *L* and *R* are the cavity width and dimensionless radius of the cylinder, respectively.

#### The Thermophysical Properties of the Hybrid Nanoliquid 

Table 2 presents the correlations used to definite the thermophysical properties of the hybrid nanoliquid [3].

### 2.2. Boundary Conditions

Table 3 lists the boundary conditions used in the presented study.

#### The Total Entropy Generation $S_{tot}$

The total entropy generation $S_{tot}$ is determined in dimensionless form as follows [23]:

(6) $$S_{tot}=S_{ht}+S_{ff}+S_{mf}$$

where *S_ht_, S_ff_, S_mf_* are the entropy generation due to the heat transfer, fluid shear, and magnetic force, respectively. Each one is expressed as follows:

(7) $$S_{ht}=\frac{k_{hnf}}{k_{f}}\left[{\left(\frac{\partial \theta }{\partial X}\right)}^{2}+{\left(\frac{\partial \theta }{\partial Y}\right)}^{2}+{\left(\frac{\partial \theta }{\partial Z}\right)}^{2}\right]$$

(8) $$\begin{array}{l} S_{ff}=\frac{\mu_{hnf}}{\mu_{f}}\varphi \left[\begin{array}{l} 2{\left(\frac{\partial U}{\partial X}\right)}^{2}+2{\left(\frac{\partial V}{\partial Y}\right)}^{2}+2{\left(\frac{\partial W}{\partial Z}\right)}^{2}\\ +{\left(\frac{\partial U}{\partial Y}+\frac{\partial V}{\partial X}\right)}^{2}+{\left(\frac{\partial W}{\partial Y}+\frac{\partial V}{\partial Z}\right)}^{2}\\ +{\left(\frac{\partial U}{\partial Z}+\frac{\partial W}{\partial X}\right)}^{2}+\frac{U^{2}+V^{2}+W^{2}}{\mathrm{Da}} \end{array}\right]\\ \; \end{array}$$

and

(9) $$S_{mf}=\varphi \frac{\sigma_{hnf}}{\sigma_{fluid}}\frac{Ha^{2}}{\varepsilon }\left(W^{2}+V^{2}\right)$$

where $\varphi =\frac{\varepsilon \mu_{hnf}T_{0}}{k_{eff}}{\left(\frac{\alpha_{nf}}{L\Delta T}\right)}^{2}$ with $T_{0}=\frac{T_{h}+T_{c}}{2}=0.5$ and $\Delta T=T_{h}-T_{c}$.

Bejan number *Be* is defined as an indicator of the ratio of heat transfer irreversibility to total entropy generation. *Be* varies between 0 and 1, where if *Be* is equal to 1, the irreversibility of the heat transfer dominates; if *Be* is equal to 0, the fluid friction dominates; and if *Be* is equal to 0.5, both heat transfer and fluid friction entropy are equilibrated. The relation for the Bejan number is given below.

(10) $$Be=\frac{S_{ht}}{S_{tot}}$$

The local and average Nusselt numbers are determined as follows:

Local Nusselt:

(11) $$Nu=-\frac{k_{eff}}{k_{f}}\frac{\partial \theta }{\partial S}$$

Average Nusselt:

(12) $$\bar{Nu}=\frac{1}{S}\int_{0}^{S}Nu\;dxdz$$

## 3. Mesh Sensitivity, Computation Procedure and Code Validation 

### 3.1. Mesh Sensitivity

**Figure 2:**
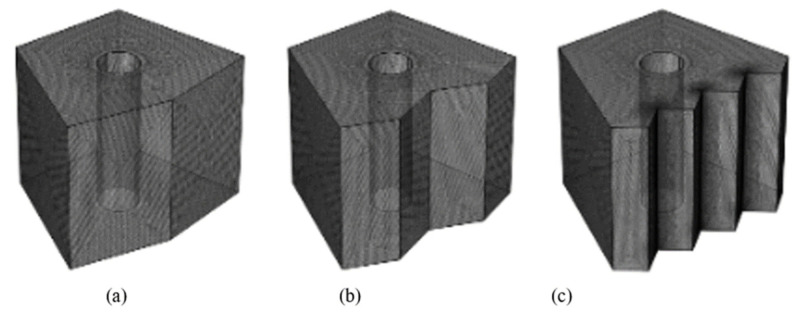
The mesh used for each configuration; (**a**) N = 1, (**b**) N = 2, and (**c**) N = 4.

For accuracy of results and economy of time, serval mesh element sizes were tested (Table 3). Figure 2 presents the mesh used for each configuration. Nusselt number was chosen as the sensitive variable, and a comparison between the abstained results for each mesh is presented in Table 4, where it can be seen that the third mesh (for the three suggested configurations) is the best choice in terms of computation time while achieving satisfactory outcomes. The grid optimization was also performed by examining the effect of the grid on computational effort in terms of CPU time. It was found that the computational effort increased by about 58% when refining the grid from 586,133 to 3,958,438.

### 3.2. Computation Procedure

The finite element method based on the Newton technique was used to discretize and solve the governing equations in a grid composed of triangular elements. The convergence of the solution is only adequate if the following convergence criterion for the relative error of each variable is achieved:

(13) $$\left|\frac{\Gamma^{i+1}-\Gamma^{i}}{\Gamma^{i+1}}\right|\leq \eta$$

where *i* indicates the iteration value and *η* represents the convergence criterion. In this numerical study, the convergence criterion was defined as *η* = 10^−6^.

### 3.3. Code Validation

**Figure 3:**
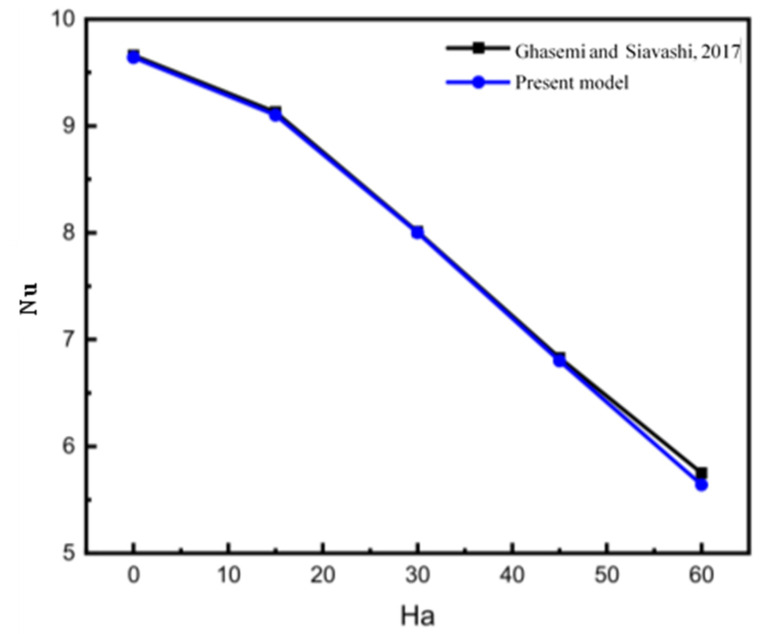
Comparison between the average Nusselt values obtained using the present model and Ghasemi and Siavashi’s results [11] for Ø = 0.4, Ra = 10^5^.

Figure 3 shows a comparison between the results obtained using the present code and the results of Ghasemi and Siavashi’s work [11]; it can be seen that there is a good agreement between the Nusselt number values of the present model and the numerical results obtained by Ghasemi and Siavashi [11].

## 4. Results and Discussion 

First of all, it should be mentioned that the value of Rayleigh number used is fixed at 10^5^ in all studied cases. 

### 4.1. The Effect of Darcy Number and the Addition of Different Concentrations of Nanoparticles on Heat Transfer and Flow Behavior 

**Figure 4:**
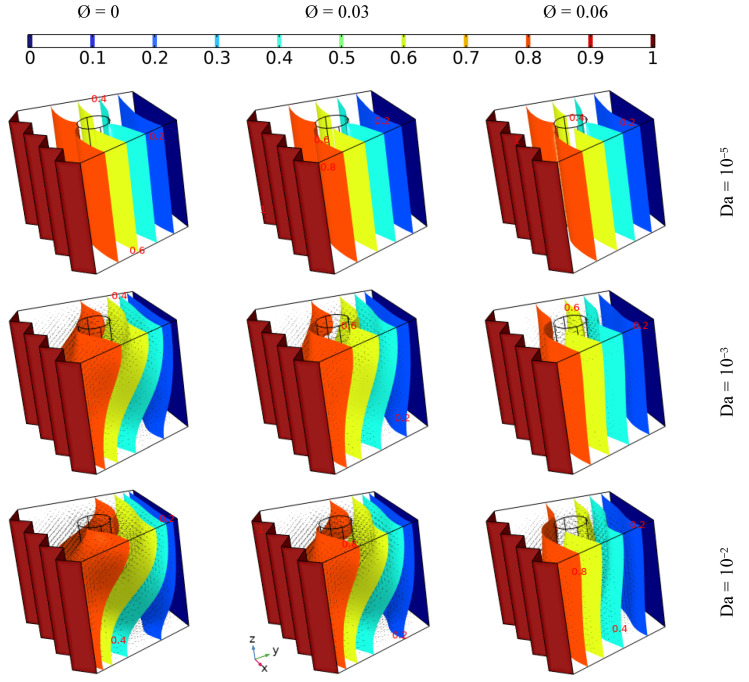
The isothermal plots and fluid patterns for different Darcy values when adding various concentrations of nanoelements with fixed angular velocity of the inner tube (Ω = 100) under no magnetic force (Ha = 0).

Figure 4 illustrates the influence of permeability (Darcy = 10^−5^, 10^−3^, and 10^−2^) in addition to different concentrations of hybrid nanoelements (from 0 to 0.06) with fixed the inner tube angular velocity (Ω = 100) and under no magnetic force (Ha = 0) on the heat distribution and fluidity inside the cavity, where the thermal progression and the flow pattern of the nanofluid are presented by isothermal plots and arrows, respectively. It can be seen that no flow was observed in the case of the lowest value of Da (10^−5^), and a tiny enhancement in heat transmission was observed with increasing concentrations of nanoelements; on the other hand, the fluidity increased with increasing Da or decreasing nanoparticle amount. Additionally, it can be observed that the isotherm plots take on an irregular shape in the cases of Da = 10^−3^ and Da = 10^−2^, regardless of the amount of nano-additives; more specifically, the fluid moves in a circular fashion around the inner tube, and the strength of the fluidity increases with increasing Da or decreasing nano-additive, where the permeability has a direct relationship with the fluidity of the fluid, which is contrary to nanoparticle concentration, where an increase in the latter augments the pressure drop while in fact having a positive effect in terms of boosting the thermal conductivity in the fluid [29].

### 4.2. The Effect of the Hartman Number on Heat Transfer and Flow Behavior

**Figure 5:**
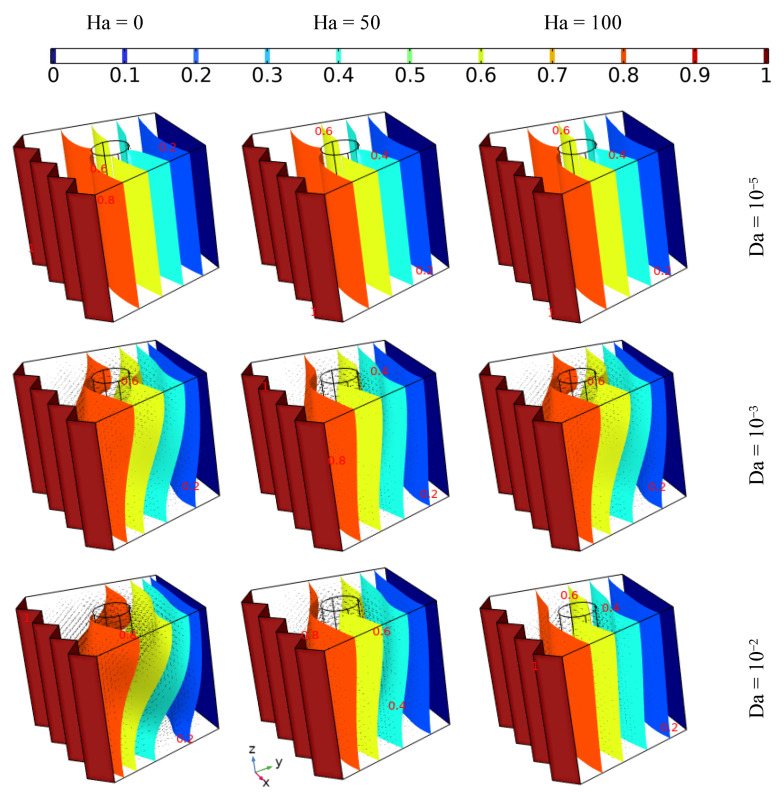
The isothermal surfaces and 3% hybrid nanofluid stream pattern with different Da and Ha values using the same tube rotation speed (Ω = 100).

The influence of employing different intensities of magnetic force (Ha = 0, 50, and 100) and different permeability values (Da = 10^−5^, 10^−3^, and 10^−2^) for the same values of inner tube velocity (Ω = 100) and the same concentrations of nanoparticles (3%) on thermal transition and flow pattern is presented in Figure 5. The results indicate that almost no fluid motion occurs in the case of Da = 10^−5^, which is in contrast to other values of Da, where fluid motion increases with increasing Da number, while the Ha value has the opposite effect on fluid fluidity due to the magnetic force causing the momentum of fluid movement to stagnate, which specifically arises due to the rotation of the inner tube. On the other hand, the isothermal surfaces are take on a more uniform shape with increasing Hartman number or decreasing Darcy number. Hence, it can be stated that the stagnation of motion is related directly to the Hartman number and inversely to the Darcy number, and that this essentially affects heat transition.

### 4.3. The Effect of the Rotation Velocity of the Cylinder on the Heat Transfer and the Flow Behavior

**Figure 6:**
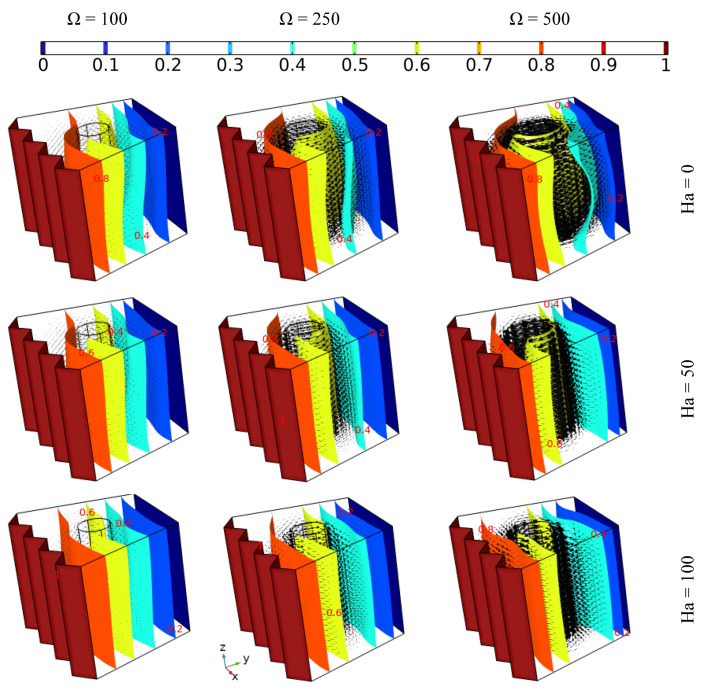
The thermal development and flow behavior of 6% hybrid nanofluid with different Hartman numbers and tube momentum speeds, using the same value of Darcy number (Da = 0.01).

Figure 6 presents the outcomes of the impact of the tube momentum speed at various values of Hartman number when using the same value of Darcy number (Da = 0.01) on the thermal development and flow behavior of 6% hybrid nanofluid. It can be observed that the intensity of the fluidity increases remarkably with increasing rotation speed of the inner tube, but with increasing value of Hartman number, the intensity of fluid motion around the tube decreases. Additionally, it should be mentioned that regardless of the fluid circulation around the tube, it can be observed that the fluid moves upwards due to the presence of the buoyancy force of free convection in the case of Ω = 100 at Ha = 0 and 50, which is in contrast to the other cases, where forced convection dominates. Furthermore, it can be concluded that the flow behavior plays an important role in the isothermal surface development, where the isothermal surfaces form an inclined shape in the case of Ω = 100 at the two lowest values of Hartman number due to free convection, which is contrast to the other cases, where the isothermal surfaces nearest to the cooled wall take on a shape parallel with it, while those nearest to the inner tube form parallel to the rounded shape, especially in the case with no magnetic force, where forced convection dominates fully.

### 4.4. The Effect of the Zigzag Pattern of the Hot Wall on the Heat Transfer and the Flow Behavior

**Figure 7:**
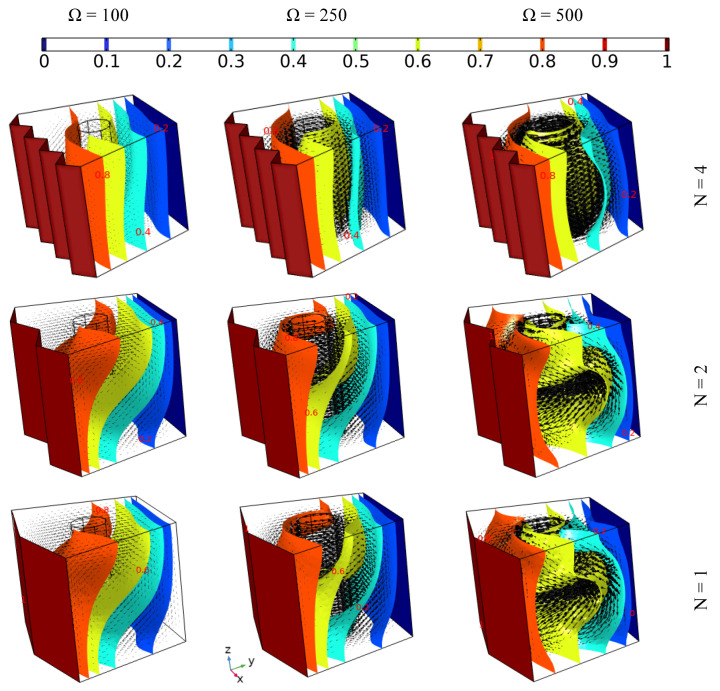
The iso-surface development and flow behavior of 6% hybrid nanofluid under various momentum speeds (Da = 0.01 and Ha = 0).

Figure 7 depicts the effect of different hot wall zigzag patterns (N = 4, 2, and 1) along with various tube rotation speeds (Ω = 100, 250, and 500) on the flow behavior and isothermal surface evolution of 6% hybrid nanofluid without applying magnetic force for Da = 0.01. The flow motion (presented by the arrows) increases with increasing tube rotation speed for all zigzag pattern cases. More specifically, in the case where Ω = 100, it can be observed that the flow circulates around the tube in an upward direction, with the main reason behind the upward motion being the buoyancy force of free convection. Thus, the shape of the isothermal surface is oblique, in contrast to the other cases where, when the tube rotation speed is higher, the isothermal surfaces take on a rounded shape beside the inner tube. Moreover, the width of the rounded shape of the isothermal surfaces is wider in the cases where N = 4, 2 than when N = 1, which can be attributed to the domination of the forced convection mode. Additionally, the intensity of the flow motion around the tube is higher in the cases where N = 4, 2 than when N = 1, which demonstrates that the zigzag pattern affects the motion and heat distribution of the fluid.

### 4.5. The Effect of Nanoparticle Amount and Darcy and Hartman Numbers on the Evolution of the Average Nusselt Number 

**Figure 8:**
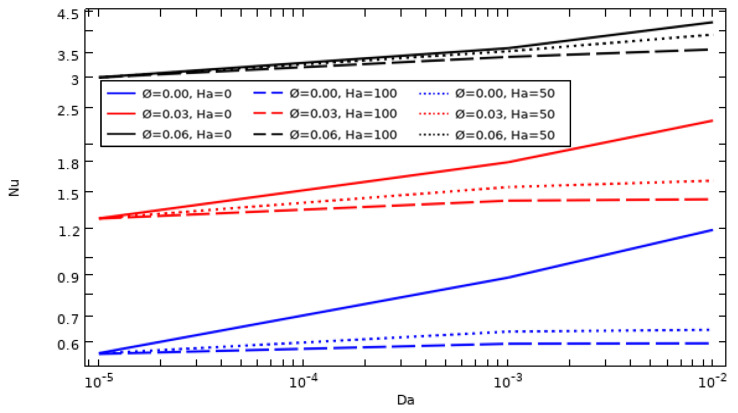
Average Nusselt number evolution as a function of Da, Ha, and nanoparticle concentration (Ω = 100).

The curves in Figure 8 present the additive effect of the Darcy number when adding different amounts of the hybrid nanocompound on the average Nusselt number $\bar{Nu}$ obtained with a hot wall without the application of magnetic force (Ha = 0) for Ω = 100. $\bar{Nu}$ improves with both increasing amount of hybrid nanoparticles and increasing Darcy number, as it is known that Nu expresses the efficacy of the heat evacuation of the system. The cooling performance is directly related to the amount of nano-additives, which improves the thermal conductivity of the host fluid; however, the cooling performance decreases at lower values of Darcy number, where the latter factor causes the fluidity of the fluid to stagnate, resulting in a degradation in heat transfer by forced convection.

### 4.6. The Effect of Cylinder Rotation Speed and Darcy and Hartman Numbers on the Evolution of the Average Nusselt Number 

**Figure 9:**
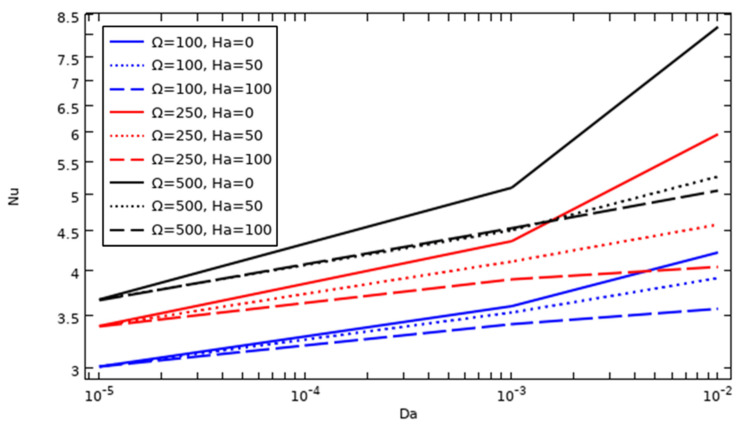
The average Nusselt number evolution as a function of tube rotation speed, Da and Ha (Ø = 0.06).

The influence of tube rotation speed and the Hartman and Darcy numbers, considering a nanoparticle concentration of 6%, on $\bar{Nu}$ value is illustrated in Figure 9. The average Nusselt number reaches high values when the tube angular velocity is increased with decreasing Ha, which means that heat dissipation can be improved by increasing the forced convection resulting from the lowest Ha value and the highest tube rotation speed.

### 4.7. The Effect of Cylinder Rotation Speed, Hartman Number, and Hot Wall Zigzag Pattern on the Evolution of the Average Nusselt Number 

**Figure 10:**
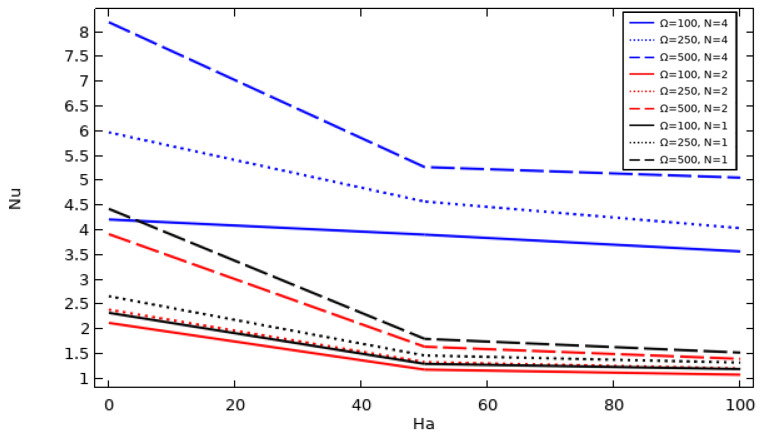
The average Nusselt number at different Ha and tube momentum speeds using different hot wall zigzag patterns (Da = 0.01 and Ø = 0.06).

Figure 10 displays the values of the average Nusselt number $\bar{Nu}$ on the hot wall (case of Ø = 0.06) for each of the cavity hot wall structures (N = 4, 2, and 1) when applying different values of Hartman number and tube momentum speed. It is well known that the Nusselt number describes the effectiveness of the heat dissipation in a system by convection. From the curves, it is clear that $\bar{Nu}$ is better when the Hartman number decreases in all studied cases. Additionally, the best improvement in cooling performance can be observed when using a hot wall structure of N = 4, followed by N = 2 and then N = 1 and with increasing inner tube rotation speed. Among the obtained results, the best $\bar{Nu}$ was achieved in the case of N = 4/Ha = 0/W = 500. 

### 4.8. The Effect of Darcy Number and Adding Different Concentrations of Nanoparticles on Bejan Number Development

**Figure 11:**
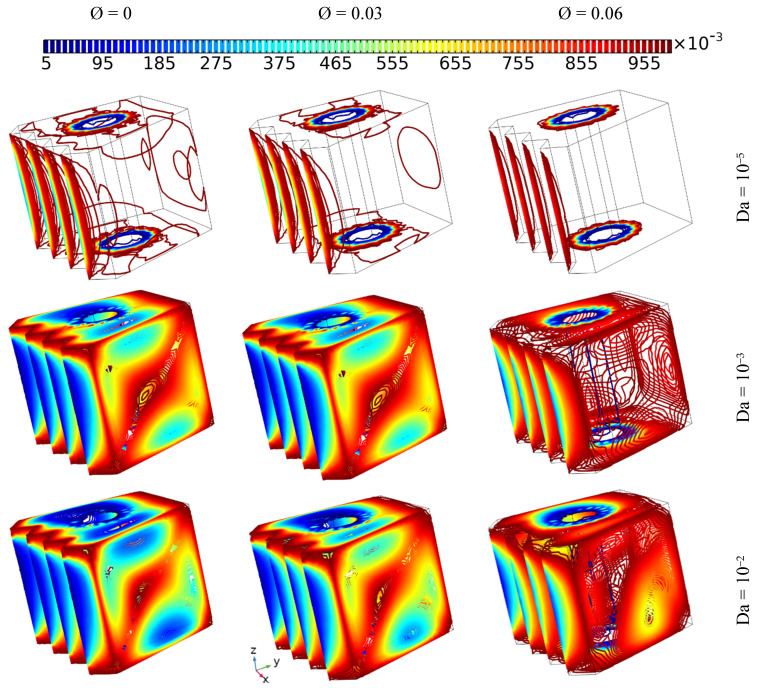
The Bejan number contours for different Darcy values and the addition of various nanoelement concentrations with fixed inner tube angular velocity (Ω = 100) and no magnetic force (Ha = 0).

Figure 11 presents the development of the Bejan number when adding different concentrations of nanoparticles (0, 0.03, and 0.06) at Da = 10^−5^, 10^−3^, and 10^−2^ for the same cylinder rotating speed (Ω = 100) without the presence of a magnetic field. As is well known, the Bejan number is the ratio of the entropy generation caused by heat transfer to the total entropy generation (caused by both heat transfer and fluid friction), where this number describes the participation of heat transfer as opposed to fluid friction; on the other hand, it is a factor aids in decreasing the irreversibility due to heat transfer. 

From the results, it can be seen that the Bejan number contours appear intensively with increasing Darcy number within the cavity due to increase in heat transfer throughout the entire cavity resulting from the increment in fluid fluidity, but it can be observed that the values of Bejan number become lower, especially close to both the hot wall and the cylinder, when the value of Da is higher, indicating that the irreversibility caused by heat transfer decreases with increasing fluid fluidity. On the other hand, the Bejan number takes on high values near the hot wall at higher solid volume fraction of nanoparticles, because the heat transfer caused by conduction is higher at the higher nanoparticle concentration. Furthermore, the high amount of these particles augments the fluid viscosity; consequently, the motion of the fluid becomes heavier, regardless of the permeability value (Darcy number).

### 4.9. The Effect of Darcy and Hartman Numbers on Bejan Number Development

**Figure 12:**
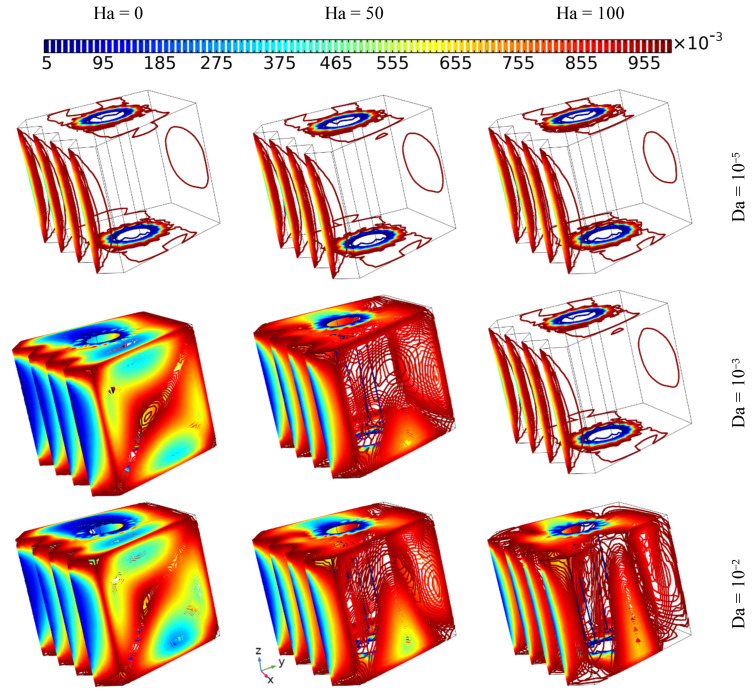
The Bejan number contours for different Da and Ha values with the same tube rotation speed (Ω = 100).

Figure 12 depicts the effect of varying Da and Ha (Da = 10^−5^, 10^−3^, and 10^−2^, Ha = 0, 50, and 100) on Bejan number when considering the same cylinder rotation speed and the addition of 3% nanoparticles. It can be observed that the contours of the Bejan number spread significantly through the cavity when lessening the magnetic force for Da = 10^−3^ and Da = 10^−2^ because the fluidity of the fluid increases with decreasing Ha and increasing Da; therefore, the spread of heat is wider due to the forced convection. At the same time, the Bejan number reaches lower values, especially close to the hot wall and the spinning tube, and a tiny reduction can be observed in the case of Da = ^−5^ when Ha is higher. Furthermore, it can be observed that Be is affected by the fluidity and the friction rate within the fluid, whereas Be decreases with increasing fluid fluidity.

### 4.10. The Effect of the Hartman Number and Cylinder Angular Velocity on Bejan Number Development

**Figure 13:**
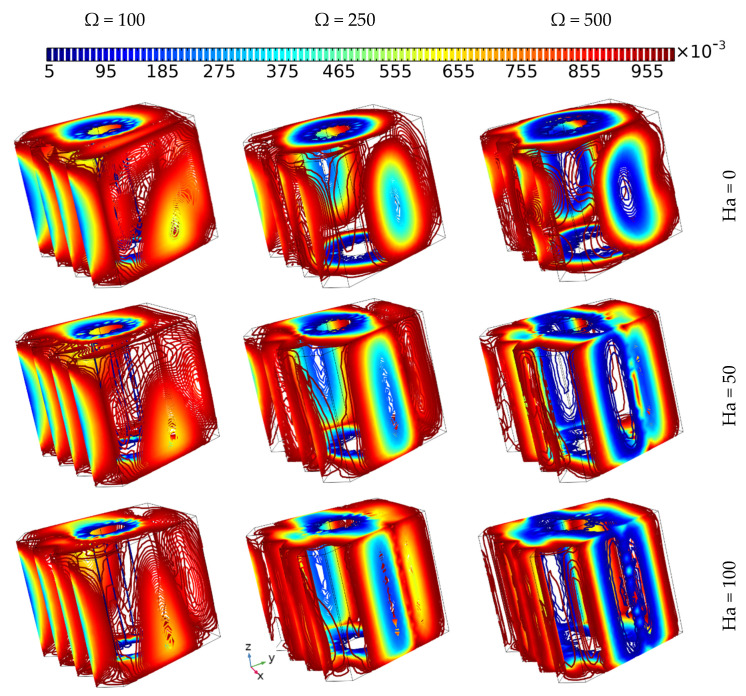
The Bejan number contours of 6% hybrid nanofluid for various Hartman numbers and tube momentum speeds, using the same value of Darcy number (Da = 0.01).

Figure 13 shows the evolution of the Bejan number when applying various magnetic forces (Ha = 0, 50, and 100) and different cylinder angular velocities (Ω = 100, 250, and 500) for Da = 10^−2^ and a nanoparticle concentration of 6%. The Bejan number contours can be observed throughout the whole cavity in all cases due to the strong fluidity resulting the present Darcy (Da^−2^) and the active effect of the rotating cylinder, which causes the heat to spread extensively (by forced convection). However, the values of Be decrease when Ha reaches low values with increasing inner tube angular velocity, especially around the cylinder, where the lowest values of Bejan number can be observed in the case of Ω = 500 at Ha = 0, as indicated by the blue lines

### 4.11. The Effect of Hot Wall Number of Undulations and Cylinder Angular Velocity on Bejan Number Development

**Figure 14:**
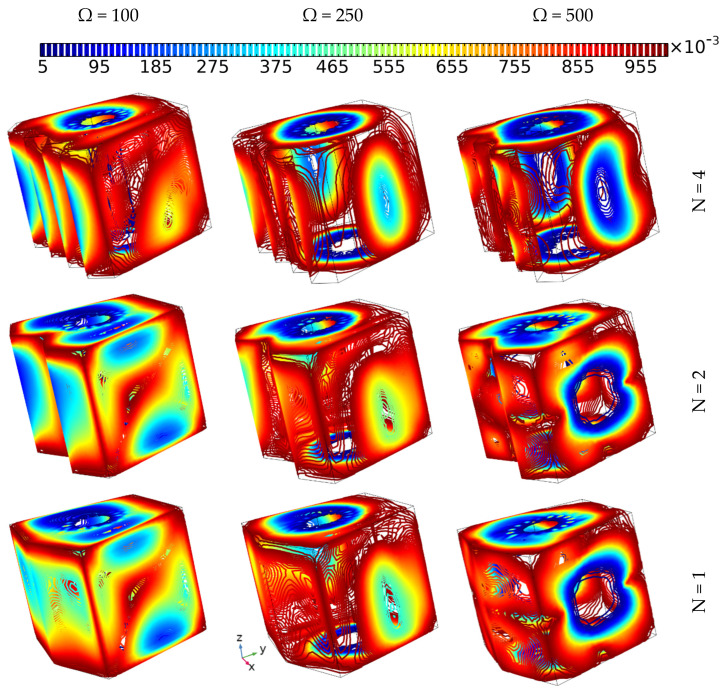
The Bejan number contours of 6% hybrid nanofluids under various momentum speeds and various hot wall undulation patterns (Da = 0.01 and Ha = 0).

Figure 14 shows the effect of the number of undulations of the hot wall (N = 1, 2, and 4) on the evolution of the Bejan number when applying different cylinder angular velocities (Ω = 100, 250, and 500) for Da = 10^−2^ and a 6% nanoparticle concentration. No visible differences can be observed between the cases where the numbers of undulations on the hot wall were set at N = 1 and N = 2, but when comparing the results of these two cases with the case where N = 4, it can be observed that at the latter number of undulations (N = 4), the Bejan number has the lowest values, regardless of the values of the angular velocity, where the latter factor always reduces the irreversibility due to heat transfer as long as the velocity is higher than that described in the above sections.

### 4.12. The Effect of the Nanoparticle Concentration and Darcy and Hartman Numbers on the Development of the Average Bejan Number 

**Figure 15:**
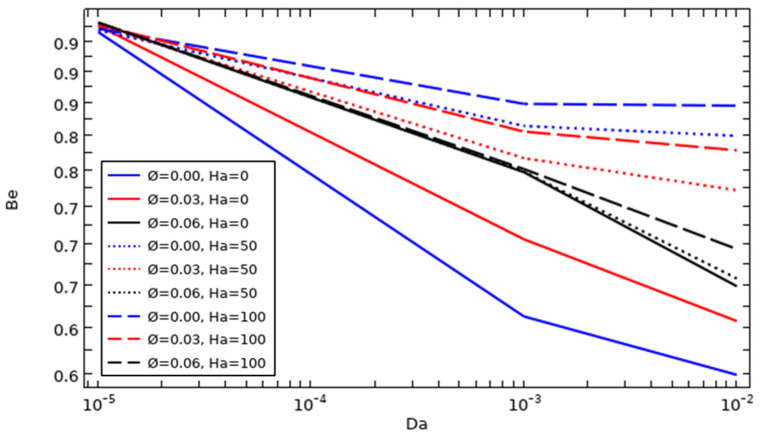
The evolution of the average Bejan number as a function of Da, Ha, and nanoparticle concentration (Ω = 100).

The curves in Figure 15 present the impact of the Darcy and Hartman numbers with the addition of different amounts of hybrid nanocompounds on the average Bejan number for Ω = 100. $\bar{Be}$ is affected by increases in the hybrid nanoparticle concentration and well as in Hartman and Darcy numbers. It is known that the Bejan number depicts the ratio of entropy generation caused by heat transfer to that caused by fluid friction. By increasing the permeability and decreasing the strength of the magnetic force, the Be decreases due to the increase in flow friction at high permeability values; in other words, the entropy that is generated by the flow friction has its highest values with high fluid motion, which leads to irreversibility due to heat transmission slightly decreasing. Increasing the amount of nanoparticles increases the thermal conductivity, but on the other hand, it also negatively affects the fluid motion, because the fluid viscosity increases, so that the entropy generation caused by heat transfer is increased and the entropy generation caused by fluid friction decreases, which is why the lowest $\bar{Be}$ can be found in the case of Ø = 0/Ha = 0/Da = 0.01.

### 4.13. The Effect of the Tube Rotation Speed and Darcy and Hartman Numbers on the Development of the Average Bejan Number 

**Figure 16:**
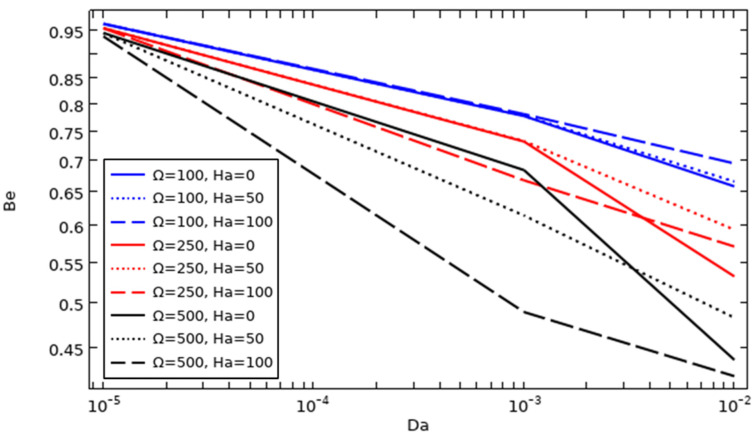
The evolution of the average Bejan number as a function of tube rotation speed, Da and Ha (Ø = 0.06).

The effect of the tube rotation speed and Darcy and Hartman numbers with 6% nanoparticle concentration on average Bejan number values is illustrated in Figure 16. The average Bejan number drops to lower values with faster tube rotation. This means that the entropy generation due to flow friction increases due to the increase in fluid motion resulting from the lowest value of Ha and the highest tube rotation speed.

More specifically, at $\mathrm{Da}\leq 10^{-3}$, the $\bar{Be}$ values are approximately the same for Ω = 100/all Ha values and Ω = 250/Ha = 0 and 50, but at Da = 0.01, the lowest values of $\bar{Be}$ are found with Ha = 0, followed by Ha = 50 and then Ha = 100 for Ω = 100, and with Ha = 0, followed by Ha = 100 and then Ha = 50 for Ω = 250. 

For Ω = 500, the lowest values of $\bar{Be}$ are found with Ha = 100, followed by Ha = 500 and then Ha = 0 when $\mathrm{Da}\leq 10^{-3}$, and at Da = 0.01, the lowest values of $\bar{Be}$ are obtained with Ha = 100, followed by Ha = 0 and then Ha = 50.

### 4.14. The Effect of the Tube Rotation Speed, the Hot Wall Zigzag Pattern, and the Hartman Number on the Development of the Average Bejan Number 

**Figure 17:**
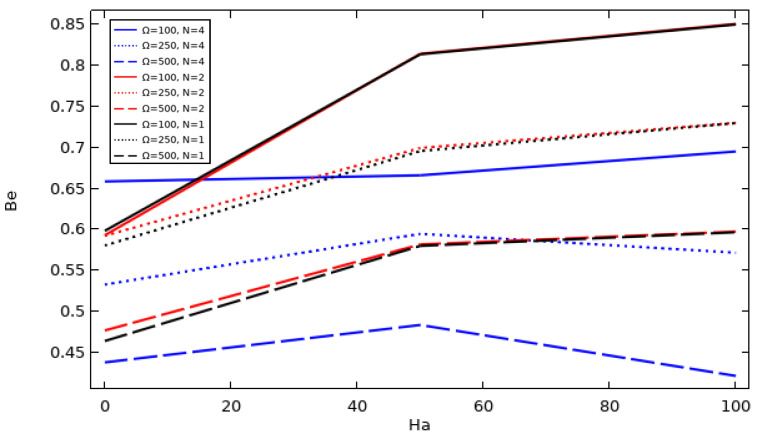
The average Bejan number for different values of Ha and tube speed with different hot wall zigzag patterns (Da = 0.01 and Ø = 0.06).

Figure 17 shows the evolution of the average Bejan number for Ø = 0.06, Da = 10^−2^ and various magnetic field intensities and inner tube rotation speeds for three different hot wall undulation patterns. It can be observed that the evolution of the average Bejan number versus Ha and Ω is a little bit complex and has different stages; thus, it is better to discuss each case separately.

A slight incensement in $\bar{Be}$ can be observed for Ω = 500/N = 1 and N = 2 with increasing Ha values, with $\bar{Be}$ values for N = 2 being superior to the case of N = 1 when Ha is below 50, following which the $\bar{Be}$ values of the two cases are equal. The same can be found for the cases with Ω = 250/N = 1, 2 and Ω = 500/N = 1, 2, but it should be noted that $\bar{Be}$ decreases with increasing inner tube rotation speed. Additionally, in the cases where Ω = 500/N = 4 and Ω = 250/N = 4, $\bar{Be}$ increases with increasing Ha until Ha = 50, but after this value, the inverse result can be observed; on the other hand, a tiny increase can be observed in $\bar{Be}$ with increasing Hartman number in the case where Ω = 100/N = 4. Finally, the case where Ω = 500/N = 4 can be found to be the best choice in terms of irreversibility. 

## 5. Conclusions 

The impact of varying the Darcy and Hartman numbers and nano solid fractions (from 0 to 6%) and utilizing various zigzag patterns of the hot wall on the heat transfer rate, flow behavior, and entropy generation of a hybrid nanofluid filled into a porous trapezoidal cavity with a spinning inner tube (at different rotating speeds Ω = 100, 250 and 500) were considered in this work.

On the basis of the interpretation of the results, the following conclusions can be drawn:

- The fluidity of the fluid is stronger with increasing Darcy number and rotating velocity of the inner tube.
- High values of Hartman number result in a significant stagnation of flow motion.
- Increasing the concentration of nanoparticles leads to an increase in the thermal conductivity of the fluid; hence, the heat spread reaches high values.
- The maximum values of average Nusselt number were reached with high numbers of zigzag undulations in the hot wall with the lowest values of Hartman number.
- The Darcy number and the rotating speed of the inner tube positively affect Nu and heat transfer rate at high values.
- The average Nusselt number increases with greater domination of forced convection, while forced convection increases with increasing inner tube spinning rate and Darcy number when there is no magnetic field.
- The entropy generation due to fluid friction is related to fluid fluidity. It increases with both increasing Darcy number and increasing angular velocity of the inner cylinder or with both decreasing Hartman number and decreasing nanoparticle amount.
- The Bejan number decreases with both increasing Darcy number and increasing angular velocity of the inner cylinder or with decreasing Hartman number and nanoparticle amount.
- High numbers of undulations in the hot wall play an important role in reducing the irreversibility phenomenon by increasing the fluid movement inside the cavity (i.e., increasing the friction in the fluid).

## Figures and Tables

**Table 1:** The thermophysical properties of water, MoS_2_, and G.O. [3].

Property	*Water*	*MoS* *_2_*	*GO*
density,ρ (kg·m^−3^)	997.1	5060	1800
Heat capacity, *C_p_* (J·kg^−1^·K^−1^)	4179	397.21	717
Thermal conductivity, k (W·m^−1^·K^−1^)	0.613	904.4	5000
Coefficient of thermal expansion, β (K^−1^)	5.8 × 10^−4^	2.8424 × 10^−5^	2.84 × 10^−4^
Electrical conductivity,σ(Ω^−1^·m^−1^)	9.75 × 10^−4^	2.09 × 10^4^	6.30 × 10^7^
Dynamic viscosity, *μ* (Pa.s)	0.001003	-	-

**Table 2:** The thermophysical properties of the hybrid nanoliquid.

Property	Classical Nanofluid	Hybrid Nanofluid
Density	$\rho_{nf}=\rho_{f}\left(1-Ø+Ø\frac{\rho_{np}}{\rho_{f}}\right)$	$\rho_{hnp}=\rho_{f}(1-{Ø}_{OG})\left((1-{Ø}_{MoS_{2}})+{Ø}_{MoS_{2}}\left(\frac{\rho_{MoS_{2}}}{\rho_{f}}\right)\right)+{Ø}_{OG}\rho_{OG}$
Heat capacity	${\left(\rho c_{p}\right)}_{nf}={\left(\rho c_{p}\right)}_{f}\left(1-Ø+Ø\frac{{\left(\rho c_{p}\right)}_{np}}{{\left(\rho c_{p}\right)}_{f}}\right)$	${\left(\rho c_{p}\right)}_{hnf}={\left(\rho c_{p}\right)}_{f}(1-{Ø}_{GO})\left((1-{Ø}_{MoS_{2}})+{Ø}_{MoS_{2}}\frac{{\left(\rho c_{p}\right)}_{MoS_{2}}}{{\left(\rho c_{p}\right)}_{f}}\right)+{Ø}_{GO}{\left(\rho c_{p}\right)}_{GO}$
Coefficient of thermal expansion	${\left(\rho \beta \right)}_{nf}={\left(\rho \beta \right)}_{f}\left(1-Ø+Ø\frac{{\left(\rho \beta \right)}_{np}}{{\left(\rho \beta \right)}_{f}}\right)$	${\left(\rho \beta \right)}_{hnf}={\left(\rho \beta \right)}_{f}(1-{Ø}_{OG})\left((1-{Ø}_{MoS_{2}})+{Ø}_{MoS_{2}}\frac{{\left(\rho \beta \right)}_{MoS_{2}}}{{\left(\rho \beta \right)}_{f}}\right)+{Ø}_{OG}{\left(\rho \beta \right)}_{OG}$
Electrical conductivity	$\frac{\sigma_{nf}}{\sigma_{f}}=1+\frac{3\left(\sigma_{np}-1\right)Ø}{\left(\sigma_{np}+2\right)-\left(\sigma_{np}-1\right)Ø}$	$\begin{array}{l} \frac{\sigma_{hnp}}{\sigma_{f}}=1+\\ \frac{3Ø({Ø}_{{}_{MoS_{2}}}\sigma_{MoS_{2}}+{Ø}_{GO}\sigma_{{}_{GO}}-\sigma_{f}({Ø}_{{}_{MoS_{2}}}+{Ø}_{GO}))}{\left({Ø}_{{}_{MoS_{2}}}\sigma_{MoS_{2}}+{Ø}_{GO}\sigma_{{}_{GO}}+2Ø\sigma_{f})-Ø\sigma_{f}(({Ø}_{{}_{MoS_{2}}}\sigma_{MoS_{2}}+{Ø}_{GO}\sigma_{{}_{GO}})-\sigma_{f}({Ø}_{{}_{MoS_{2}}}+{Ø}_{GO})\right)} \end{array}$
Thermal conductivity	$\frac{k_{nf}}{k_{f}}=\frac{k_{np}+(n-1)k_{f}-(n-1)Ø(k_{f}-k_{np})}{k_{np}+(n-1)k_{f}+Ø(k_{f}-k_{np})}$	$\frac{k_{hnf}}{k_{bf}}=\frac{k_{GO}+(n-1)k_{bf}-(n-1){Ø}_{GO}(k_{bf}-k_{GO})}{k_{GO}+(n-1)k_{bf}+{Ø}_{GO}(k_{bf}-k_{GO})}$ $\mathrm{where},\frac{k_{bf}}{k_{f}}=\frac{k_{MoS_{2}}+(n-1)k_{f}-(n-1){Ø}_{MoS_{2}}(k_{f}-k_{MoS_{2}})}{k_{MoS_{2}}+(n-1)k_{f}+{Ø}_{MoS_{2}}(k_{f}-k_{MoS_{2}})}$
Viscosity	$\mu_{nf}=\frac{\mu_{f}}{{\left(1-Ø\right)}^{2.5}}$	$\mu_{hnp}=\frac{\mu_{f}}{{\left(1-{Ø}_{MoS_{2}}\right)}^{2.5}{\left(1-{Ø}_{OG}\right)}^{2.5}}$
$Ø={Ø}_{MoS2}+{Ø}_{OG}$ $,\;{Ø}_{MoS2}={Ø}_{OG}=\frac{Ø}{2}$		

*nf*: nanofluid, *np*: nanoparticle.

**Table 3:** The boundary conditions.

	Thermal Condition	Velocity Condition
The inclined Lift wall	Adiabatic	U,V,W=0 (no-slip)
The inclined right wall	adiabatic	U,V,W=0 (no-slip)
The top wall	adiabatic	U,V,W=0 (no-slip)
The Bottom wall	adiabatic	U,V,W=0 (no-slip)
The zigzag wall	θ=1	U,V,W=0 (no-slip)
The front wall of the zigzag one	θ=0	U,V,W=0 (no-slip)
The central tube wall	adiabatic	U=−Ω(Y−OY), V=Ω(X−OX)

**Table 4:** Grid sensitivity tests (Ø = 0.03, Ω = 100, Ha = 0 and Da = 0.01).

Total Number of Elements		Average Nusselt Number
N = 4	137,688	2.0245
N = 2	69,639	1.7521
N = 1	69,180	2.0041
N = 4	348,525	2.2758
N = 2	182,971	1.8745
N = 1	181,506	2.0940
N = 4	994,968	2.3167
N = 2	590,354	1.9595
N = 1	586,133	2.1472
N = 4	3,958,438	2.3174
N = 2	2,542,173	1.9599
N = 1	2,519,898	2.1502

## Data Availability

Not applicable.

## Nomenclatures

**Symbols**	
*B*	Magnetic field
*Be*	Bejan number
*c_p_*	Specific heat (J·kg^−1^·K^−1^)
Da	Darcy number
*g*	Gravitational acceleration, (m·s^−2^)
Ha	Hartman number
*k*	Thermal conductivity, (W·m^−1^·K^−1^)
*L*	Cavity width, (m)
*Nu*	Nusselt number
*p*	Pressure, (Pa)
*P*	Dimensionless pressure
Pr	Prandtl number
*R*	The dimensionless radius of the cylinder
*r*	The radius of the cylinder
*Ra*	Rayleigh number
*Re*	Reynolds number
*Ri*	Richardson number
*S*	Entropy generation
*T*	Temperature, (K)
*u, v, w*	Velocities in x, y, z-directions, (m·s^−1^)
*U, V, W*	Dimensionless velocities component in X, Y, Z-directions
*X, Y, Z*	Dimensionless coordinates in x, y, and z directions
**Greek letters**	
α	Thermal diffusivity (m^2^·s^−1^)
β	Thermal expansion coefficient (K^−1^)
ε	Porosity
θ	Dimensionless temperature
κ	Permeability (m^2^)
μ	Dynamic viscosity (Pa.s)
ν	Kinematic viscosity (m^2^·s^−1^)
ρ	Density (kg/m^3^)
σ	Electrical conductivity (Ω^−1^·m^−1^)
ϕ	Nanoparticle concentration
Ω	Dimensionless angular rotational velocity
ω	Angular rotational velocity (rad·s^−1^)
**Subscript**	
*eff*	Effective
*f*	fluid
*ff*	Fluid friction
*hnf*	Hybrid nanofluid
*mf*	Magnetic field
*nf*	Nanofluid
*np*	Nanoparticle
*s*	Solid matrix
*th*	Thermal
*tot*	Total
